# The clinical value of artificial intelligence in assisting junior radiologists in thyroid ultrasound: a multicenter prospective study from real clinical practice

**DOI:** 10.1186/s12916-024-03510-z

**Published:** 2024-07-12

**Authors:** Dong Xu, Lin Sui, Chunquan Zhang, Jing Xiong, Vicky Yang Wang, Yahan Zhou, Xinying Zhu, Chen Chen, Yu Zhao, Yiting Xie, Weizhen Kong, Jincao Yao, Lei Xu, Yuxia Zhai, Liping Wang

**Affiliations:** 1 Department of Diagnostic Ultrasound Imaging & Interventional Therapy, Zhejiang Cancer Hospital, Hangzhou Institute of Medicine (HIM), Chinese Academy of Sciences, Hangzhou, 310022 China; 2 Wenling Institute of Big Data and Artificial Intelligence in Medicine, Taizhou, 317502 China; 3 Zhejiang Provincial Research Center for Cancer Intelligent Diagnosis and Molecular Technology, Hangzhou, 310022 China; 4 Taizhou Key Laboratory of Minimally Invasive Interventional Therapy & Artificial Intelligence, Taizhou Campus of Zhejiang Cancer Hospital (Taizhou Cancer Hospital), Taizhou, 317502 China; 5 Department of Ultrasound, The Second Affiliated Hospital, Jiangxi Medical College, Nanchang University, Nanchang, 330006 China; 6 Institute of Biomedical and Health Engineering, Shenzhen Institutes of Advanced Technology, Chinese Academy of Sciences, Shenzhen, 518055 China; 7 Demetics Medical Technology Co. Ltd., Hangzhou, 310022 China; 8 Department of Mathematics, The University of Hong Kong, Hong Kong, 999077 China; 9 Zhejiang Qiushi Institute for Mathematical Medicine, Hangzhou, 310022 China; 10 Present address: Division of Medical Imaging and Technology, Department of Clinical Science, Intervention and Technology, Karolinska Institutet, Stockholm, Sweden; 11 The Second Affiliated Hospital of Shantou University Medical College, Guangdong, 515041 China

**Keywords:** Thyroid nodule, Ultrasound, Artificial intelligence, Diagnosis criteria

## Abstract

**Background:**

This study is to propose a clinically applicable 2-echelon (2e) diagnostic criteria for the analysis of thyroid nodules such that low-risk nodules are screened off while only suspicious or indeterminate ones are further examined by histopathology, and to explore whether artificial intelligence (AI) can provide precise assistance for clinical decision-making in the real-world prospective scenario.

**Methods:**

In this prospective study, we enrolled 1036 patients with a total of 2296 thyroid nodules from three medical centers. The diagnostic performance of the AI system, radiologists with different levels of experience, and AI-assisted radiologists with different levels of experience in diagnosing thyroid nodules were evaluated against our proposed 2e diagnostic criteria, with the first being an arbitration committee consisting of 3 senior specialists and the second being cyto- or histopathology.

**Results:**

According to the 2e diagnostic criteria, 1543 nodules were classified by the arbitration committee, and the benign and malignant nature of 753 nodules was determined by pathological examinations. Taking pathological results as the evaluation standard, the sensitivity, specificity, accuracy, and area under the receiver operating characteristic curve (AUC) of the AI systems were 0.826, 0.815, 0.821, and 0.821. For those cases where diagnosis by the Arbitration Committee were taken as the evaluation standard, the sensitivity, specificity, accuracy, and AUC of the AI system were 0.946, 0.966, 0.964, and 0.956. Taking the global 2e diagnostic criteria as the gold standard, the sensitivity, specificity, accuracy, and AUC of the AI system were 0.868, 0.934, 0.917, and 0.901, respectively. Under different criteria, AI was comparable to the diagnostic performance of senior radiologists and outperformed junior radiologists (all *P* < 0.05). Furthermore, AI assistance significantly improved the performance of junior radiologists in the diagnosis of thyroid nodules, and their diagnostic performance was comparable to that of senior radiologists when pathological results were taken as the gold standard (all* p* > 0.05).

**Conclusions:**

The proposed 2e diagnostic criteria are consistent with real-world clinical evaluations and affirm the applicability of the AI system. Under the 2e criteria, the diagnostic performance of the AI system is comparable to that of senior radiologists and significantly improves the diagnostic capabilities of junior radiologists. This has the potential to reduce unnecessary invasive diagnostic procedures in real-world clinical practice.

**Supplementary Information:**

The online version contains supplementary material available at 10.1186/s12916-024-03510-z.

## Background

Thyroid nodules are very common in clinical practice, with ultrasound (US) detection rates as high as 65% in the general population [1]. Though most thyroid nodules are benign and malignant nodules smaller than 1 cm frequently exhibit nonaggressive behavior, the mortality of thyroid cancer increases with a rate of 0.6% per year from 2009 to 2018 [2]. Effective and noninvasive screening of malignant thyroid nodules from benign ones is highly desirable in the clinics. There exist a number of Thyroid Imaging, Reporting, and Data Systems (TI-RADS) all defined in five categorical features of composition, echogenicity, shape, margin, and echogenic foci but with subtle differences, for thyroid nodule risk stratification [3]. These TI-RADS criteria provide reliable and noninvasive US screening guidelines for thyroid nodules. However, the accuracies in malignancy differentiation are highly dependent on radiologists’ personal experience levels and subjective judgments, resulting in significant intra- and inter-observer variations [4].

The development of data-driven AI algorithms on the premise of a sufficiently large and well-balanced training dataset has enabled diagnostic efficacies that match or may even surpass those of senior radiologists, providing radiologists with an objective second opinion for predicting the malignant status of thyroid nodules [5]. In clinical studies, it is common to assess the diagnostic efficacies of an AI model taking the postoperative pathology as the gold standard [6–8]. To date, there is no real methodological alternative to the postoperative pathology for the final diagnosis of thyroid nodules. The drawback however is that for the sake of ethical concerns, thyroidectomy is typically performed for diagnostic purposes if the associated thyroid nodules are considered to have stratified risk levels. The direct consequence of defining the gold standard as such is that easily discernible nodule cases would have to be removed from evaluation studies as the postoperative pathological evidence is lacking. This unavoidably distorts the sampling distribution for diagnostic efficacy evaluation and introduces systematic biases against individual raters. A practical alternative is to take minimally invasive fine needle aspiration cytology (FNAC) as a complimentary gold standard for those cases that do not meet the criteria for taking postoperative pathological examinations (PPE) [9, 10]. This however can introduce two weaknesses, one being that not all cases are subjected to FNAC as they still have to fulfill certain criteria defined by US risk stratification systems (RSSs) [11], and the other being that for cases that use FNAC results as the gold standard, according to the Bethesda risk stratification system [12] definition standard, the ultimate diagnoses are in fact uncertain for Bethesda categories III and IV, making an absolute evaluation of each individual rater’s diagnostic efficacy difficult. In spite of the second weakness, FNAC may be practically used as a standard for cases comprising Bethesda categories II and VI [5] from the perspective of ethical concerns such that over-treatment should be minimized as much as possible without simultaneously incurring a substantial sacrifice of reliability for malignancy diagnosis [13]. As FNAC is not without risks [14] and not recommended for every nodule case, for cases diagnosed to fall out of Bethesda categories II and VI, PPE is needed to set the final diagnosis. As such, the most pragmatic definition of diagnostic evaluation standard in the perspective clinical scenario shall follow the practical workflow in which the TI-RADS criteria, FNAC, and PPE all play their individual indispensable roles in the diagnostic processes. Following this principle, in this research study, we did not exclude cases simply because the corresponding diagnoses were not performed as in many cases for the sake of allowing the diagnostic performance evaluation using their defined evaluation standard. All cases that passed our quality checks were included. However, due to the lack of a sufficient number of nodule samples which were classified to be Bethesda II and VI categories after FNAC, we grouped these cases together with the cases finally diagnosed by PPE, forming our proposed 2e diagnostic criteria for diagnostic efficacy evaluation of different groups, meaning that all nodules which were considered unnecessary for further FNAC or PPE diagnosis took the decision of an arbitration group of 3 senior specialists when referring to the TI-RADS criteria as the final diagnosis, otherwise the pathology-based diagnosis was taken as the gold standard.

In this study, we evaluated the diagnostic performance of an AI system, AI-SONIC™ Thyroid with an algorithm named US_THYROID_S, version A1.01.001.001 (Demetics Medical Technology, Ltd.), for malignant thyroid nodule screening on prospectively collected US images of the patients with thyroid nodules in three first-tier research-intensive hospitals located in three different provincial regions. The purpose of this study is to analyze thyroid nodules using the 2e criteria that are in line with clinical practice, to investigate whether AI can provide precise assistance, especially for junior radiologists in clinical decision-making.

## Methods

### Patients

This prospective study was approved by the local ethical committee of each medical center. Written informed consent was obtained from each patient prior to undergoing US examinations. Patients were continuously admitted to three medical centers, i.e., The Cancer Hospital of the University of Chinese Academy of Sciences (Medical Center 1), The Second Affiliated Hospital of Shantou University (Medical Center 2), and The Second Affiliated Hospital of Nanchang University (Medical Center 3) for thyroid nodule examinations. The US examinations were carried out following a previous guideline [11] using central frequency in the range of 5–10 MHz with Colour Doppler US machines. Details of US machines are supplemented in Additional file 1: Table S1.

The needed sample size for this study was estimated using the equation [15] for a one-sided test:

$$N=\frac{{[{Z}_{1-a}\sqrt{{P}_{0}(1-{P}_{0})}+{Z}_{1-\beta }\sqrt{{P}_{T}(1-{P}_{T})}]}^{2}}{{({P}_{T}-{P}_{0})}^{2}},$$

in which *P*_*T*_ represents the expected sensitivity or specificity, *P*_*0*_ represents a clinically acceptable lower bound for sensitivity or specificity, *Z*_1*-α*_ is the normal deviate at 1-*α* confidence level, and *Z*_1*- β*_ is the normal deviate at 1-*β* power, while *α* and *β* represent the probability of type I and type II errors respectively. The expected sensitivity and specificity for the AI system were 90% and 85% while the targeted sensitivity and specificity were 85% and 80%. For a confidence level of 95% and power of 80%, assuming a loss of 20% during data collection, 363 positive and 471 negative cases were needed.

The data collection started from November 2, 2021, and ended on February 21, 2022, to fulfill the need for sample size.

### Inclusion and exclusion criteria

**Fig. 1:**
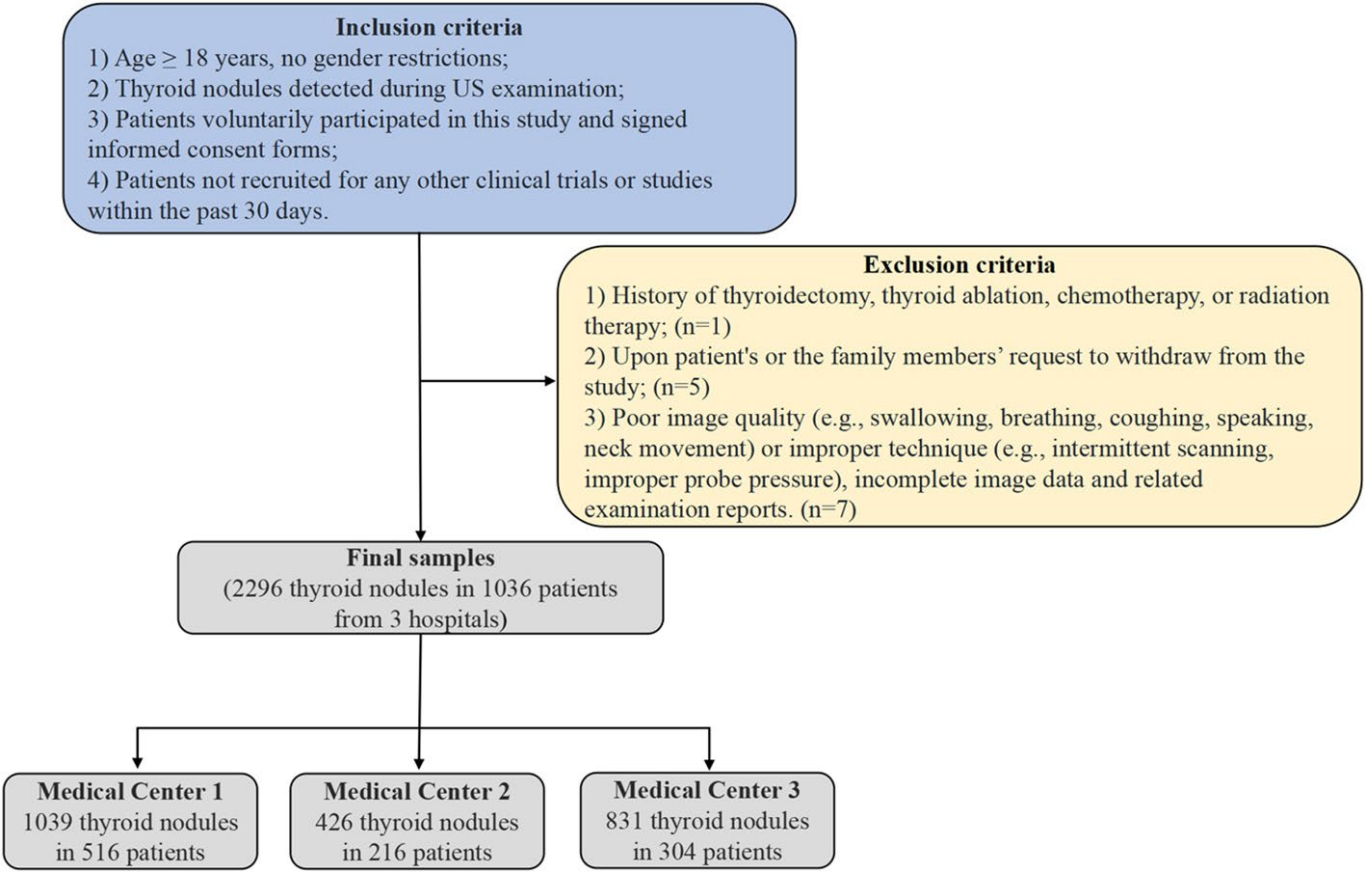
Flowchart of the study

From November 2, 2021, to February 21, 2022, 1040 consecutive patients with 2309 thyroid nodules who underwent thyroid US examination at three medical centers were initially enrolled. Only patients with nodules who met all of the following criteria (Fig. 1) were included in this clinical study:

1. Age ≥ 18 years, no gender restrictions;
2. Thyroid nodules detected during US examination;
3. Patients voluntarily participated in this study and signed informed consent forms; and
4. Patients not recruited for any other clinical trials or studies within the past 30 days.

However, patients were excluded from this study if they met the following criteria:

1. History of thyroidectomy, thyroid ablation, chemotherapy, or radiation therapy ( *n* = 1);
2. Upon the patient’s or the family members’ request to withdraw from the study (*n* = 5); and
3. Poor image quality (e.g., swallowing, breathing, coughing, speaking, neck movement) or improper technique (e.g., intermittent scanning, improper probe pressure), incomplete image data, and related examination reports (*n* = 7).

A total of 2296 thyroid nodules from 1036 patients were finally included for analysis.

### Acquisition and quality control of US images

In this study, a total of 5 radiologists (2 radiologists from Medical Center 1, 1 radiologist from Medical Center 2, and 2 radiologists from Medical Center 3) acquired US images following their respective hospital’s US examination protocol. After US image acquisitions, these radiologists selected the most representative transverse and longitudinal planes of each nodule, de-identified and serialized the images, manually segmented regions of interests (ROIs) around the target nodules and subsequently transmitted the original US images and ROIs to the AI system for display and analysis. This is done not because the AI system is not capable of segmenting the target nodules, but to eliminate the compounding effect of mixing nodule segmentation with diagnostic performance, as we focused primarily on evaluating its diagnosis capability.

Based on the original images and supplied ROIs, the AI system provided its US diagnostic recommendations with binary predictions (potentially benign/malignant) using convolutional neural network deep learning technology. Grayscale US images, segmented masks along with the binary diagnostic results provided by the AI system were then stored in the database.

For quality control, all US images included for data analysis and diagnostic efficacy evaluation should fulfill the requirement for clinical diagnostic usage following the AIUM practice guideline for performing thyroid US [16]. The images were stored without local magnification in the format of BMP, PNG, TIF, JPG/JPEG, or uncompressed DICOM with an image size of at least 150 KB, containing ≥ 640 * 480 pixels. The images should not be too blurred to evaluate the ultrasonographical features of the thyroid nodules. The nodular features should not be seriously corrupted by imaging artifacts and the gain settings should provide adequate contrast for nodular feature evaluation. There should be no measuring marks or texts in the interior or around the peripheries of the nodules that disturbed the interpretation of images. The nodules should be well positioned within the field of view unless they were too big to fit in.

### US diagnosis by AI system and radiologists

The AI software is developed on the EfficientNet architecture [17] using a proprietary deep learning framework DE-Light. Typically, such an AI system returns a predicted malignant probability value for each nodule in the US image, ranging from 0 to 1, allowing users to customize the cut-off value for performance optimization using for instance a retrospective dataset. However, for this study, the AI system supplied by the manufacturer which came with its own dedicated hardware that could directly connect to a US machine had a fixed internal cut-off value of 0.6, predetermined using their internal retrospective test dataset. If the probability value was ≥ 0.6, a nodule was considered as malignant, otherwise benign. It is important to note that the internally computed malignancy probability was not exposed to users for the supplied AI system in this study, which differed from previous retrospective clinical studies using AI systems provided by the same manufacturer [18–20]. As a result, the area under the receiver operating characteristic curve (AUC) value for evaluating the diagnostic results by the AI system was calculated using the binary predictions of potentially benign or malignant but not continuous values between 0 and 1. This change predated the initialization of this clinical study and it was an internal decision made solely by the manufacturer.

Every 3 months, US reports excluding the AI’s diagnostic predictions from three medical centers were sent separately to an evaluation group of four radiologists, who were requested to make a binary classification of pro-benign or pro-malignant according to the ACR-TIRADS criteria and their own clinical experiences independently. Among the four radiologists, two were junior radiologists with ≥ 3 but ≤ 5 years of US diagnostic experience, and two were senior radiologists with ≥ 10 years of US diagnostic experience, all recruited from the participating hospitals. In addition, an arbitration committee consisting of three senior radiologists with more than 15 years in thyroid US examinations was also recruited from The General Hospital of the People’s Liberation Army, The First Affiliated Hospital of Zhejiang University, and The Cancer Hospital of the University of Chinese Academy of Sciences respectively. Two committee members received a copy of the reports and made their interpretations independently. Only when disagreements between them occurred, their diagnostic reports were sent to the third committee member for an arbitration; otherwise, their consensus set the final decision for US-based diagnosis.

Six months after all diagnoses on recruited patients were completed, the 4 radiologists who participated in this study repeated their diagnoses on US images however with consulting the AI system.

It is important to note that the ACR-TIRADS criteria do not lead to binary classifications as its purpose is not to make binary diagnosis but to grade the malignancy risk levels and provide suggestions for clinical management. However, as personal experience plays a vital role for radiologists’ decision-making process, and there was no consensus guideline about setting a categorical cutoff according to ACR-TIRADS categories, besides that the aim of this study was not to evaluate whose categorical classifications complied better with the malignancy probability distribution underlying the ACR-TIRADS criteria, it was decided in this study that radiologists evaluated malignancy-relevant attributes according to the ACR-TIRADS criteria but provided a binary assessment on whether the nodule was more probable to be benign or malignant in combination with their personal experiences.

### The 2e diagnostic criteria

Our diagnostic criteria for thyroid nodule diagnosis were designed to take the form of a two-level hierarchy, with the first being the decision from an Ultrasonography Review and Arbitration Committee by referring to the ACR TI-RADS and their own clinical experiences, the second being the pathological result (FNAC or PPE). The details of the 2e diagnostic criteria established are as follows.

**Fig. 2:**
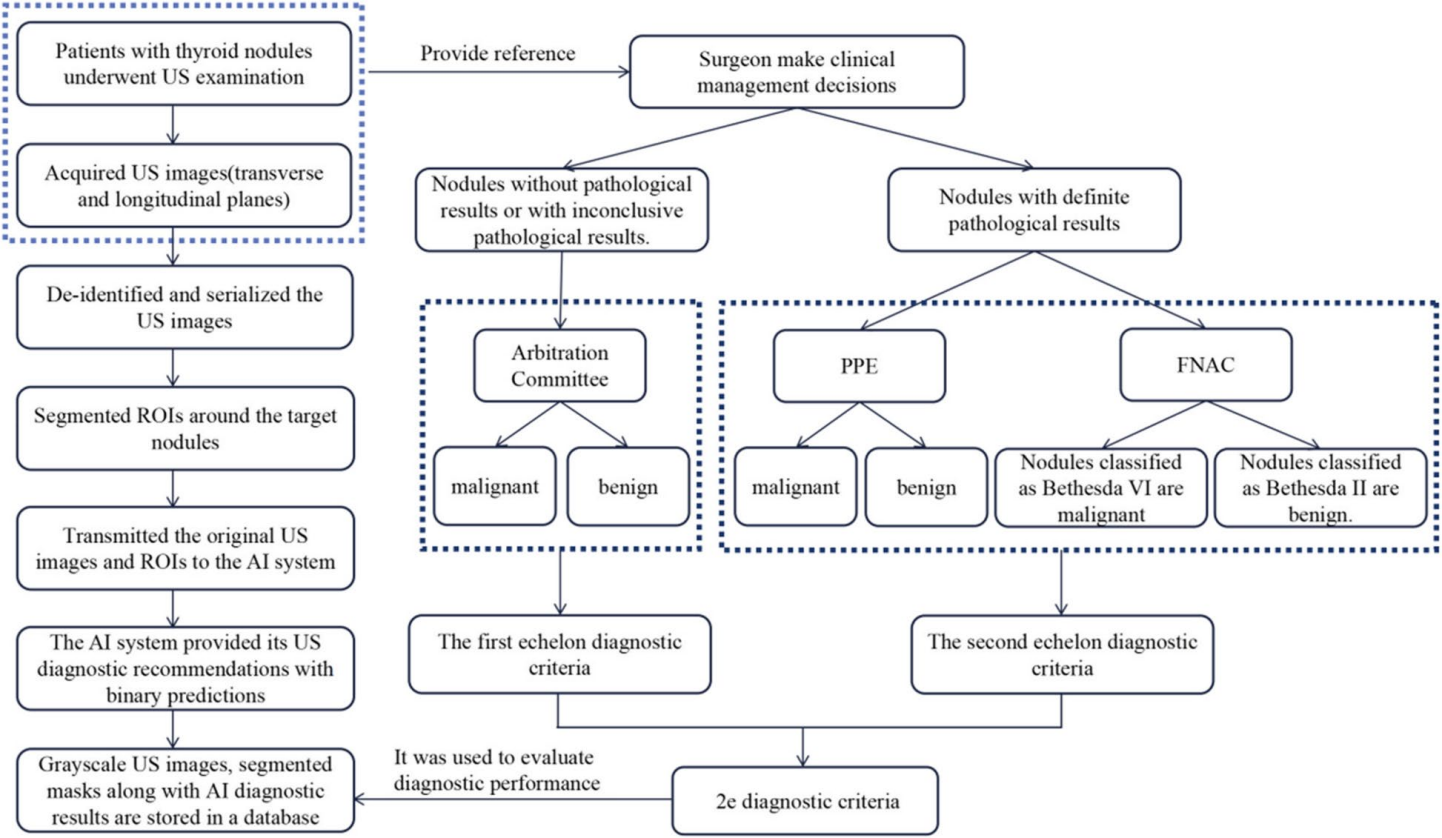
Flowchart of the 2e diagnostic criteria

For nodules without pathological results, namely nodules that did not require further intervention with reference to ultrasonographic findings (clearly benign, or suspicious for malignancy but did not meet the criteria for biopsy), and nodules with inconclusive pathological results (not classified as Bethesda II or VI after FNAC and without PPE), the consensus of the review committee was used as the standard. In case FNAC was performed, a nodule of the Bethesda II category was diagnosed as benign while as malignant if classified as the Bethesda VI category according to the Bethesda System for Reporting Thyroid Cytopathology (2017 Edition) [21]. Otherwise, PPE diagnosis served as the gold standard for assessing the benign or malignant nature. This detailed flowchart is supplemented in Fig. 2. All clinical management decisions regarding patients with thyroid nodules were made by surgeons according to the 2015 American Thyroid Association guideline [22] and guidelines for the diagnosis and management of thyroid nodules and differentiated thyroid cancer (Second edition) [23].

### Statistical analyses

Descriptive statistics were used to report the patient’s age, sex distribution, and benign and malignant nodules determined by the 2e diagnostic criteria. Age was described as the mean and standard deviation, while the classification results confirmed by each diagnostic method were described as both absolute numbers and percentages calculated relative to each evaluation standard. In addition, to compare the diagnostic efficacy of the AI system with that of the junior radiologists as well as that of the radiologists under the assistance of AI, we calculated the sensitivity, specificity, and accuracy together with their 95% confidence intervals as well as the AUC. In addition, the *Z*-value test was used to compute *P*-values for statistical comparisons between different groups for evaluation metrics other than the AUC values, whose comparison was done instead using the DeLong test [24]. We followed the convention of considering a *P*-value less than 0.05 as statistically significant. Statistical analysis was performed using Python 3.8 (Python Software Foundation, DE, USA).

## Results

### Sample statistics

Table 1 summarizes the basic sample statistics of patient age, gender, and the number of benign and malignant nodules determined through US examination arbitration committee, FNAC, and PPE at each medical center. A total of 1036 patients were recruited for this study (516 patients from Medical Center 1, 216 patients from Medical Center 2, and 304 patients from Medical Center 3), with a total of 2296 nodules. Among them, there were 260 male patients and 776 female patients. According to the 2e diagnostic criteria, a total of 1707 nodules were diagnosed as benign: among which 1339 nodules were diagnosed by the US examination arbitration committee, 368 nodules were diagnosed by pathological result (28 nodules were diagnosed by FNAC, and 340 nodules were diagnosed by PPE). There were 589 malignant nodules, with 204 nodules diagnosed by the US examination arbitration committee and 385 nodules diagnosed by pathological result (11 nodules diagnosed by FNAC, and 374 nodules diagnosed by PPE).

**Table 1:** Summary of clinical features of patients and thyroid nodule

Characteristics	Medical Center 1	Medical Center 2	Medical Center 3
**Number of patients**	516	216	304
**Age (mean ± SD)**	51.44 ± 12.32 (20.36, 83.39)	46.52 ± 13.50 (18.43, 74.72)	45.73 ± 12.74 (19.62, 83.28)
**Sex**			
Male	140 (27.1%)	55 (25.5%)	65 (21.4%)
Female	376 (72.9%)	161 (74.5%)	239 (78.6%)
**Number of nodules**	1039	426	831
**Location of nodules**			
Isthmus	60	19	52
Lateral lobe	979	407	779
**Benign nodules**	752 (72.4%)	347 (81.5%)	608 (73.2%)
Arbitration Committee	638 (84.8%)	222 (64.0%)	479 (78.8%)
FNAC	8 (1.1%)	14 (4.0%)	6 (1.0%)
PPE	106 (14.1%)	111 (32.0%)	123 (20.2%)
**Malignant nodules**	287 (27.6%)	79 (18.5%)	223 (26.8%)
ACR TI-RADS	97 (33.8%)	36 (45.6%)	71 (31.8%)
FNAC	6 (2.1%)	1 (1.3%)	4 (1.8%)
PPE	184 (64.1%)	42 (53.2%)	148 (66.4%)

*SD* standard deviation, *FNAC* fine needle aspiration cytology, *PPE* postoperative pathological examinations

### Pathological result as the evaluation standard

In this study, the benign and malignant nature of 753 nodules was determined by pathological result (among which 714 nodules were determined by PPE), with 368 nodules classified as benign and 385 nodules classified as malignant. For these thyroid nodules, the AI system demonstrated comparable sensitivity, specificity, accuracy, and AUC to senior radiologists (0.826 vs. 0.800, 0.815 vs. 0.804, 0.821 vs. 0.802, 0.821 vs. 0.802, respectively; all *p* > 0.05). The specificity, accuracy, and AUC of the AI system were superior to that of less experienced junior radiologists (0.815 vs. 0.701, 0.821 vs. 0.745, 0.821 vs. 0.744, respectively; all *p* < 0.001). Compared to independent readings by junior radiologists, AI-assisted readings significantly improved their specificity, accuracy, and AUC (all *p* < 0.05). We found that AI-assisted junior radiologists could achieve the diagnostic level of senior radiologists (all *p* > 0.05) (Table 2).

**Table 2:** Diagnostic performance of AI, individual radiologists, and AI-assisted radiologists taking pathological result as the evaluation standard

**Method**	**Pathological result**		**Sensitivity** **95% CI**	***p* value**	**Specificity** **95% CI**	***p* value**	**Accuracy** **95% CI**	***p* value**	**AUC** **95% CI**	***p* value**
	**Malignancy**	**Benign**								
**AI**										
Malignancy	318	68	0.826 (0.788, 0.864)		0.815 (0.776, 0.855)		0.821 (0.793, 0.848)		0.821 (0.793, 0.848)	
Benign	67	300								
**Junior radiologists**										
Malignancy	303	110	0.787 (0.746, 0.828)	0.055*	0.701 (0.654, 0.748)	< 0.001*	0.745 (0.714, 0.776)	< 0.001*	0.744 (0.713, 0.775)	< 0.001*
Benign	82	258								
**Senior radiologists**										
Malignancy	308	72	0.800 (0.760, 0.840)	0.196*	0.804 (0.764, 0.845)	0.591*	0.802 (0.774, 0.831)	0.356*	0.802 (0.774, 0.831)	0.361*
Benign	77	296								
**Junior radiologists + AI**										
Malignancy	307	74	0.797 (0.757, 0.838)	0.619^	0.799 (0.758, 0.840)	** < 0.001**^	0.798 (0.769, 0.827)	**0.014^**	0.798 (0.769, 0.827)	**0.012^**
Benign	78	294		0.900^#^		0.791^#^		0.847^#^		0.845^#^
**Senior radiologists + AI**										
Malignancy	305	64	00.792 (0.752, 0.833)	0.707^	0.826 (0.788, 0.865)	0.277^	0.809 (0.781, 0.837)	0.745^	0.809 (0.781, 0.837)	0.732^
Benign	80	304								

*CI* confidence interval, *AUC* area under the receiver operating characteristic curve, *AI* artificial intelligence

^*^p values to compare radiologists with the AI system

^*p* values to compare radiologists with and without assistance of AI

^#^*p* values to compare junior radiologists with assistance of AI to senior radiologists alone

In addition, the benign and malignant nature of 714 nodules was determined by PPE. For these thyroid nodules, the AI system demonstrated comparable performance to experienced radiologists in terms of sensitivity, specificity, accuracy, and AUC (all *P* > 0.05). The specificity, accuracy, and AUC of the AI system were superior to those of less experienced radiologists (all *P* < 0.05). The AI-assisted mode significantly improved the specificity, accuracy, and AUC of less experienced radiologists to the level of senior radiologists, while the sensitivity remained similar to their independent readings (Additional file 1: Table S2). To validate the high diagnostic performance of the US examination arbitration committee in determining the benign and malignant nature of thyroid nodules, sensitivity, specificity, accuracy, and AUC were calculated using PPE as the gold standard, resulting in values of 0.789, 0.865, 0.825, and 0.827, respectively (Additional file 1: Table S2).

### Diagnosis by the Arbitration Committee as the evaluation standard

In this study, 1543 nodules were classified by the arbitration committee without proceeding to FNAC or PPE diagnosis, with 1339 nodules classified as pro-benign and 204 nodules classified as pro-malignant. For these thyroid nodules, the AI system demonstrated excellent agreement to senior radiologists in terms of sensitivity, specificity, accuracy, and AUC (0.946 vs. 0.941, 0.966 vs. 0.970, 0.964 vs. 0.966, 0.956 vs. 0.956, respectively; all *p* > 0.05). The AI system showed superior sensitivity, specificity, accuracy, and AUC compared to that of junior radiologists (all *p* < 0.001). Compared to independent readings by junior radiologists, AI-assisted readings significantly improved their specificity, accuracy, and AUC (0.875 vs. 0.955, 0.876 vs. 0.943, 0.878 vs. 0.909, respectively; all *p* < 0.05), but did not reach the level of senior radiologists (all *p* < 0.05) (Table 3).

**Table 3:** Diagnostic performance of AI, individual radiologists, and AI-assisted radiologists evaluated against the diagnosis by the Arbitration Committee

**Method**	**Arbitration Committee**		**Sensitivity** **95% CI**	***p* value**	**Specificity** **95% CI**	***p* value**	**Accuracy** **95% CI**	***p* value**	**AUC** **95% CI**	***p* value**
	**Malignancy**	**Benign**								
**AI**										
Pro-malignancy	193	45	0.946 (0.915, 0.977)		0.966 (0.957, 0.976)		0.964 (0.954, 0.973)		0.956 (0.946, 0.966)	
Pro-benign	11	1294								
**Junior radiologists**										
Pro-malignancy	180	168	0.882 (0.838, 0.927)	< 0.001*	0.875 (0.857, 0.892)	< 0.001*	0.876 (0.859, 0.892)	< 0.001*	0.878 (0.862, 0.895)	< 0.001*
Pro-benign	24	1171								
**Senior radiologists**										
Pro-malignancy	192	40	0.941 (0.909, 0.973)	0.555*	0.970 (0.961, 0.979)	0.554*	0.966 (0.957, 0.975)	0.695*	0.956 (0.945, 0.966)	0.937*
Pro-benign	12	1299								
**Junior radiologists + AI**										
Pro-malignancy	176	60	0.863 (0.816, 0.910)	0.102^	0.955 (0.944, 0.966)	** < 0.001**^	0.943 (0.931, 0.955)	** < 0.001**^	0.909 (0.895, 0.923)	**0.006^**
Pro-benign	28	1279		** < 0.001**^#^		**0.029**^**#**^		**0.002**^**#**^		** < 0.001**^#^
**Senior radiologists + AI**										
Pro-malignancy	194	27	0.951 (0.921, 0.981)	0.228^	0.980 (0.972, 0.987)	0.084^	0.976 (0.968, 0.984)	0.107^	0.965 (0.956, 0.975)	0.164 ^
Pro-benign	10	1312								

*CI* confidence interval, *AUC* area under the receiver operating characteristic curve, *AI* artificial intelligence

^*^p values to compare radiologists with the AI system

^*p* values to compare radiologists with and without assistance of AI

^#^*p* values to compare junior radiologists with assistance of AI to senior radiologists alone

### Global 2e diagnostic criteria as the gold standard

Summing over all cases which were analyzed separately using either pathology or the arbitration committee as the diagnostic criteria, or in our proposed term, the 2e diagnostic criteria, the AI system demonstrated superior sensitivity, specificity, accuracy, and AUC compared to junior radiologists (0.868 vs. 0.820, 0.934 vs. 0.837, 0.917 vs. 0.833, 0.901 vs. 0.829, respectively; all *p* < 0.001), and showed comparable results to senior radiologists’ readings (0.868 vs. 0.849, 0.934 vs. 0.934, 0.917 vs. 0.912, 0.901 vs. 0.892, respectively; all *p* > 0.05). Compared to independent readings by junior radiologists, AI-assisted readings significantly improved their specificity, accuracy, and AUC (0.837 vs. 0.921, 0.833 vs. 0.895, 0.829 vs. 0.871, respectively; all *p* < 0.001), where specificity and accuracy were comparable to those of senior radiologists (*p* > 0.05) (Table 4).

**Table 4:** Diagnostic performance of AI, individual radiologists, and AI-assisted radiologists, evaluated against 2e diagnostic criteria

**Method**	**2e diagnostic criteria**		**Sensitivity** **95% CI**	***p* value**	**Specificity** **95% CI**	***p* value**	**Accuracy** **95% CI**	***p* value**	**AUC** **95% CI**	***p* value**
	**Malignancy**	**Benign**								
**AI**										
Malignancy	511	113	0.868 (0.840, 0.895)		0.934 (0.922, 0.946)		0.917 (0.906, 0.928)		0.901 (0.888, 0.913)	
Benign	78	1594								
**Junior radiologists**										
Malignancy	483	278	0.820 (0.789, 0.851)	< 0.001*	0.837 (0.820, 0.855)	< 0.001*	0.833 (0.817, 0.848)	< 0.001*	0.829 (0.813, 0.844)	** < 0.001**
Benign	106	1429								
**Senior radiologists**										
Malignancy	500	112	0.849 (0.820, 0.878)	0.070*	0.934 (0.923, 0.946)	0.936*	0.912 (0.901, 0.924)	0.597*	0.892 (0.879, 0.904)	0.315*
Benign	89	1595								
**Junior radiologists + AI**										
Malignancy	483	134	0.820 (0.789, 0.851)	1.000^	0.921 (0.909, 0.934)	** < 0.001^**	0.895 (0.883, 0.908)	** < 0.001^**	0.871 (0.857, 0.884)	** < 0.001^**
Benign	106	1573		**0.008**^**#**^		0.091^#^		0.051**#**		**0.029**^**#**^
**Senior radiologists + AI**										
Malignancy	499	91	0.847 (0.818, 0.876)	0.873^	0.947 (0.936, 0.957)	0.078^	0.921 (0.910, 0.932)	0.286^	0.897 (0.885, 0.909)	0.557^
Benign	90	1616								

*CI* confidence interval, *AUC* area under the receiver operating characteristic curve, *AI* artificial intelligence

^*^p values to compare radiologists with the AI system

^*p* values to compare radiologists with and without assistance of AI

^#^*p* values to compare junior radiologists with assistance of AI to senior radiologists alone

In addition, the diagnostic performance of AI was compared with that of radiologists in each of the three medical centers included in this study. In Medical Center 1, which included 1039 thyroid nodules (Additional file 1: Table S3), the AI system exhibited higher specificity, accuracy, and AUC compared to junior radiologists’ readings (0.938 vs. 0.883, 0.924 vs. 0.877, 0.913 vs. 0.872 respectively; all *p* < 0.05), but showed no significant differences compared to senior radiologists’ readings (all *p* > 0.05). Compared to independent readings by junior radiologists, AI-assisted readings improved specificity (0.883 vs. 0.939, *p* < 0.001) and accuracy (0.877 vs. 0.910, *p* = 0.013), which were comparable to those of senior radiologists (all *p* > 0.05). In Medical Center 2, which included 426 thyroid nodules (Additional file 1: Table S4), the AI system exhibited higher sensitivity, specificity, accuracy, and AUC compared to junior radiologists’ readings (all *p* < 0.05). There were no significant differences between senior radiologists and AI in terms of specificity, accuracy, and AUC (all *p* > 0.05). Compared to independent readings by junior radiologists, AI-assisted readings significantly improved their specificity, accuracy, and AUC (all *p* < 0.001), which were comparable to those of senior radiologists (all *p* > 0.05). There were no significant differences in sensitivity, specificity, accuracy, and AUC between AI-assisted and independent readings by senior radiologists (all *p* > 0.05). In Medical Center 3, which included 831 thyroid nodules (Additional file 1: Table S5), the AI system exhibited significantly higher sensitivity, specificity, accuracy, and AUC compared to junior radiologists (all *p* < 0.001), but comparable results to experienced radiologists’ readings (all *p* > 0.05). Furthermore, AI assistance led to an improved overall diagnostic AUC for junior radiologists (*p* < 0.05).

**Fig. 3:**
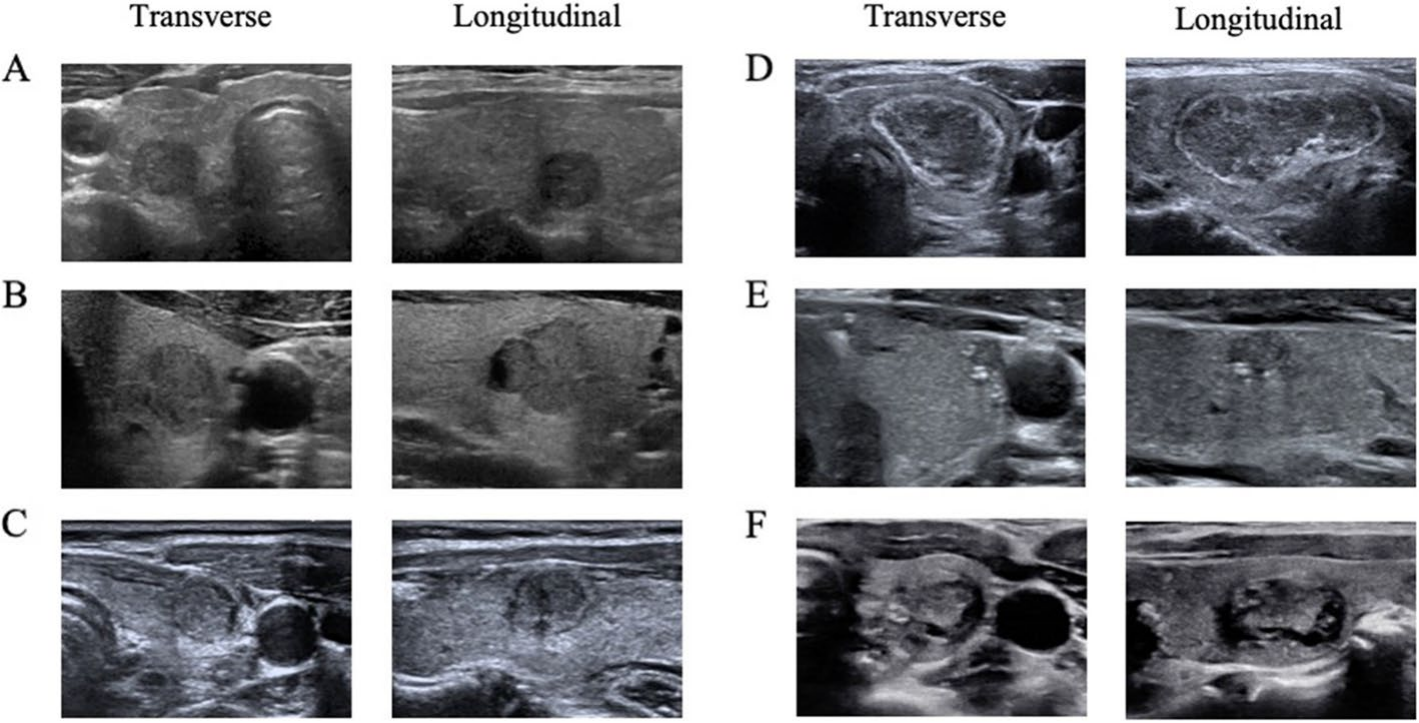
Representative transverse and longitudinal US images of thyroid nodules selected from six different cases, covering miss-diagnosis of AI and senior radiologists, success and failure in assisting junior radiologists to achieve better diagnosis according to our proposed 2e diagnosis criteria. **A** Both the AI system and senior radiologists diagnosed it as “malignant,” and the PPE was nodular goiter with adenomatous hyperplasia. **B** Both the AI system and senior radiologists classified it as “benign,” but it was diagnosed to be papillary thyroid microcarcinoma according to the PPE. **C**, **D** The AI system diagnosed as “malignant” and junior radiologists independently diagnosed as “benign,” which was however changed to “malignant” after referring to the AI. **C** was diagnosed as thyroid micropapillary carcinoma, and **D** as Hashimoto’s thyroiditis with thyroid adenoma according to the PPE. **E**, **F** The AI system diagnosed as “benign” and junior radiologists independently diagnosed as “malignant,” which was changed to “benign” after referring to the AI. **E** was diagnosed as papillary thyroid microcarcinoma, while **F** did not undergo pathological examination and was considered as “benign” by the arbitration committee

However, out of 2296 nodules in total except cases where junior radiologists corrected their original miss-classifications of nodule malignancy after consulting recommendations of the AI system (32 and 161 respective nodule cases for which originally miss-classified as benign or malignant were corrected), there were also cases where diagnosis by the AI system misled the junior radiologists (17 and 14 respective nodule cases for which originally correctly-classified as benign or malignant were reverted). In addition, there were also cases where the AI system failed simultaneously as the senior radiologists (81 and 59 respective nodule cases where benign and malignant nodules were miss-classified) according to our proposed 2e diagnostic criteria(Additional file 1: Table S6). We selected representative thyroid nodule US images from these cases shown in Fig. 3 which might provide some hints about the decision-makings from radiologists and the AI system.

It is also interesting to evaluate whether diagnosis by the AI system could also be helpful for the junior radiologists in diagnosing isthmic nodules. This is defined as such if the transverse US image of the thyroid nodule is entirely or mostly located in front of the trachea, or in other words in the isthmus [25]. Compared with the nodules in lateral lobes of the thyroid gland, nodules located in the isthmus could pose certain diagnostic challenges. Therefore, we analyzed specifically also nodules located in the isthmus of the thyroid (Additional file 1: Table S7), revealing that the AI system significantly outperformed junior radiologists in terms of specificity and AUC (all *p* < 0.05) and also it showed a higher accuracy, however without statistical difference (*p* = 0.075). When compared to the senior radiologists, it showed effectively equivalent performance (all *p* > 0.05). Compared to independent readings by junior radiologists, AI-assisted readings improved specificity (0.795 vs. 0.918, *p* = 0.004) and AUC (0.777 vs. 0.855, *p* = 0.016).

## Discussion

In this study, utilizing the proposed 2e diagnostic criteria as the evaluation standard, we conducted a discrimination between benign and malignant thyroid nodules across three medical centers. The results demonstrated that the diagnostic performance of the AI system was comparable to that of highly experienced senior radiologists in thyroid nodule assessment using US across conventionally used evaluation metrics. The AI system improved junior radiologists’ diagnostic performances in terms of specificity, accuracy, and AUC, and their diagnostic performance was comparable to that of senior radiologists using pathological results as the gold standard. Additionally, this study performed analysis of diagnosis of thyroid nodules by radiologists of different levels and the AI system across the three medical centers using different diagnostic criteria namely pathology and consensus of senior specialists separately, yielding consistent results. Consequently, we assert that AI systems can provide specific decision support for thyroid nodule US diagnosis in real-world clinical practice across various scenarios.

It has been very common to evaluate the diagnostic performances of AI systems for cancer diagnoses using the PPE as the sole gold standard. There is no doubt that the PPE provides the final diagnosis with no methodological alternatives to-date. The problem however is that for ethical concerns, the overwhelming majority of thyroid nodules on patients are benign, and a substantial fraction of them do not need to take FNAC for diagnostic purposes, needless to mention more invasive and complication-causing thyroidectomy [26] which is necessary for PPE. Concurrently, there is a growing concern across the globe that thyroid cancers may have been over-treated [27–29]. As such, the applicability of using postoperative pathology in clinical practices as the only diagnostic gold standard is questionable. Incorporating FNAC as a complementary diagnostic standard causes less complications to patients as it is less invasive and can be used as an alternative to PPE for certain cases, namely Bethesda category II and VI. However, except for cases with inconclusive diagnosis, it still bears the blame for removing a substantial fraction of nodules from statistical analysis which do not exhibit suspicious features and therefore fail to meet the criterion for taking FNAC. Besides, holding clinicians accountable for deciding what further examinations shall be taken after US examinations of thyroid nodules would very much likely persist in the foreseeable future. In this regard, including US interpretation of radiologists to the diagnostic standard is justifiable. However, due to unavoidable intra- and inter-observer variations, setting up a group of experts to review the US examinations helps reconcile the challenge of benefiting from subjective assessment while confining its inherent weakness to the possible minimum. In this study, to assess the diagnostic performance of the expert panel, we calculated their sensitivity, specificity, accuracy, and AUC for diagnosing clearly pathological nodules after surgery. The diagnostic performance of the US examination arbitration committee was consistent with the diagnostic performance of previous thyroid US experts [9, 30]. Therefore, it is permissible to use the US examination arbitration committee's readings as the gold standard for those nodules which didn’t undergo pathological examinations by either FNAC or PPE in this study, as it aligns with actual clinical practice. Having a two-level hierarchical or in our terms 2e standard for diagnostic performance evaluation acknowledges the significant roles of experts in US as well as their limitations. Though this definition of diagnostic standard still carries its disadvantage presumably for not being ideally precise, we argue that it fits well to the clinical practices such that it reaches a good balance of being precise and minimizing sampling bias simultaneously. We expect our proposed 2e diagnostic criteria to be applicable for other diagnostic scenarios where experts’ subjective opinions complement objective criteria.

The application of AI in the field of US is becoming increasingly widespread, especially in the diagnosis of thyroid nodules [31–33]. Previous studies have mostly built AI models based on thyroid nodule US images with clear pathological results for training. However, On the one hand, many thyroid nodules diagnosed in clinical practice do not have clear pathology, and the diagnosis mainly relies on the assessment of clinical doctors, leading to differences in diagnostic results due to different clinical experiences [34]. On the other hand, the inclusion of only nodules with definite pathology that were benign accounted for only a small proportion of actual benign nodules, which would cause selection bias [35]. Therefore, considering these factors, we propose the use of the 2e diagnostic criteria as our main approach, which sets us apart from other studies. Meanwhile, our AI system still achieves good results in evaluating thyroid nodules based on this criterion. Chen et al. [36] included 636 patients with a total of 1588 thyroid nodules, and the nature of these nodules was pathologically confirmed postoperatively. They developed a multi-task deep learning model based on ACR TI-RADS features to assess the benign or malignant nature of thyroid nodules. In the test dataset, this model achieved an AUC of 0.91 and a sensitivity of 83%, surpassing the performance of junior radiologists (with AUC and sensitivity of 0.78 and 70%, respectively) but without a significant difference compared to experienced radiologists (with AUC and sensitivity of 0.93 and 92%, respectively). Furthermore, the model exhibited a specificity of 87%, which was higher than both junior and senior radiologists (with specificity of 80% and 75%, respectively). In our study, an analysis of all included thyroid nodules revealed that the sensitivity, specificity, and AUC of the AI system were superior to that of the junior radiologists (all *P* < 0.05), consistent with the results of the study by Chen et al. However, our study yielded differing results when comparing the diagnostic performance of AI and senior radiologists, with AI in our study performing equivalently to senior radiologists. This trend persisted across different medical centers and various diagnostic criteria. Li et al. [37] prospectively included 236 patients with 312 thyroid nodules, with FNAC or PPE as the gold standard. They analyzed the performance of AI, resident physicians, and senior radiologists in the diagnosis of benign and malignant nodules both with and without AI assistance. The results showed that the AI system achieved a sensitivity, accuracy, and AUC of 0.95, 0.84, and 0.753, respectively, which was on par with senior radiologists (all *P* > 0.05). Additionally, the AI-assisted strategy significantly improved the overall diagnostic performance of junior radiologists (all *P* < 0.01), aligning with the conclusions of our study. Though isthmic nodules could pose potential challenges for the AI system compared with nodules in the lateral lobes, the AI system could significantly improve junior radiologists’ diagnostic specificity and AUC value for isthmic nodules. In total, there were substantially more cases where the junior radiologists benefited from consulting diagnostic results by the AI system than being misled (193 vs 31 summing up benign and malignant classified nodules). It is noteworthy that their study used PPE results or a combination of PPE and FNAC results as the gold standard for thyroid nodule classification. Compared with others, our research has achieved favorable results under both the PPE diagnostic criteria and the 2e diagnostic criteria. This also confirms the potential wide application of the proposed 2e diagnostic criteria in AI-related studies. Furthermore, the 2e diagnostic criteria are highly applicable to clinical practice, which emphasizes the capacity of AI in providing accurate clinical decision support. Particularly for less-experienced radiologists, they can greatly enhance their diagnostic abilities with the help of AI, thus reducing unnecessary biopsies, alleviating over-diagnosis, and preventing over-treatment.

This study has several limitations. First, the process of thyroid nodule scanning by radiologists was dynamic, whereas the information provided to the AI system and radiologists for diagnosis was based on static images, resulting in a reduced set of relevant features compared to those obtainable through dynamic scanning. Second, the nature of thyroid nodules was not entirely determined by PPE. Some nodules were evaluated qualitatively through FNAC or US arbitration committee, the latter of which in fact accounted for a large fraction. The lower the TIRADS grade, the lower the probability of malignancy. A previous study [38] showed the distribution between TI-RADS classification and malignant nodules as follows: 1.1% (2/175) for TI-RADS 1, 5.3% (9/170) for TI-RADS 2, 4.1% (22/536) for TI-RADS 3, 10.6% (90/850) for TI-RADS 4, and 22.7% (49/216) for TI-RADS 5. Therefore, TIRADS is reliable as a diagnostic criterion for nodules that do not meet the criteria for pathological examinations. Finally, this study exclusively took B-mode US images of thyroid nodules and did not incorporate other multi-modal US imaging techniques, such as elastography and color Doppler imaging for nodule evaluation.

## Conclusions

The 2e diagnostic criteria we proposed align with real-world clinical assessment and affirm the universality of AI systems. Under the 2e diagnostic criteria, the diagnostic performance of AI systems is on par with that of highly experienced senior radiologists and has the potential to enhance the diagnostic capabilities of junior radiologists significantly. This, in turn, reduces unnecessary invasive diagnostic procedures and treatments for patients. The further development of AI technology is bound to have a profound impact on the thyroid nodule diagnostic process in the future.

### Supplementary Information

Additional file 1: Table S1-S7. Table S1. Details of US machines in the three medical centers. Table S2. Diagnostic performance of AI, individual radiologists, and AI-assisted radiologists taking PPE as the evaluation standard. Table S3. Medical Center 1: diagnostic performance of AI, individual radiologists, and AI-assisted radiologists, evaluated against 2e diagnostic criteria. Table S4. Medical Center 2: diagnostic performance of AI, individual radiologists, and AI-assisted radiologists, evaluated against 2e diagnostic criteria. Table S5. Medical Center 3: diagnostic performance of AI, individual radiologists, and AI-assisted radiologists, evaluated against 2e diagnostic criteria. Table S6. Six representative situations of the AI system and the radiologists in the diagnosis results. Table S7. Nodules in the thyroid isthmus: diagnostic performance of AI, individual radiologists, and AI-assisted radiologists, evaluated against 2e diagnostic criteria.

## Data Availability

The datasets analyzed during the current study are not publicly available due to the metadata containing information that could compromise the patients but are available from the corresponding author on reasonable request.

## Abbreviations

2e: 2E-echelon
AI: Artificial intelligence
AUC: Area under the receiver operating characteristic curve
CI: Confidence interval
FNAC: Fine needle aspiration cytology
PPE: Postoperative pathological examinations
ROIs: Regions of interests
RSSs: Risk stratification systems
SD: Standard deviation
TI-RADS: Thyroid Imaging, Reporting and Data Systems
US: Ultrasound
