# Supplementary material for: The clinical value of artificial intelligence in assisting junior radiologists in thyroid ultrasound: a multicenter prospective study from real clinical practice
Source: BMC Med. 2024 Jul 12;22:293. doi: 10.1186/s12916-024-03510-z (PMC11241898; doi:10.1186/s12916-024-03510-z)
Supplement: Supplementary file 1 — Additional file 1: Table S1-S7. Table S1. Details of US machines in the three medical centers. Table S2. Diagnostic performance of AI, individual radiologists, and AI-assisted radiologists taking PPE as the evaluation standard. Table S3. Medical Center 1: diagnostic performance of AI, individual radiologists, and AI-assisted radiologists, evaluated against 2e diagnostic criteria. Table S4. Medical Center 2: diagnostic performance of AI, individual radiologists, and AI-assisted radiologists, evaluated against 2e diagnostic criteria. Table S5. Medical Center 3: diagnostic performance of AI, individual radiologists, and AI-assisted radiologists, evaluated against 2e diagnostic criteria. Table S6. Six representative situations of the AI system and the radiologists in the diagnosis results. Table S7. Nodules in the thyroid isthmus: diagnostic performance of AI, individual radiologists, and AI-assisted radiologists, evaluated against 2e diagnostic criteria. [file 12916_2024_3510_MOESM1_ESM.docx]

**Supplementary materials**

Table S1 Details of US machines in the three medical centers.

| **Hospital** | **US machines** | **Number of patients** |
| --- | --- | --- |
| Medical Center 1 | GE Healthcare LOGIQE Portable Ultrasound | 51 |
|  | GE Healthcare LOGIQ E9 | 79 |
|  | Hitachi ARIETTA 70 | 89 |
|  | PHILIPS EPIQ 7 | 60 |
|  | TOSHIBA Aplio400 | 69 |
|  | Esaote mylab class c | 73 |
|  | SonoScape | 40 |
|  | Siemens | 57 |
|  | | **518** |
| Medical Center 2 | SUMSUNG RS80A | 104 |
|  | MINDRAY Resona 7S | 95 |
|  | TOSHIBA TUS-A500 | 106 |
|  | | **305** |
| Medical Center 3 | GE LOGIQ E9 | 10 |
|  | Canon i900 | 101 |
|  | Mindray Resona7 | 96 |
|  | Samsung RS80A | 4 |
|  | Samsung WS80A | 6 |
|  | | **217** |
| Total number |  | **1040** |

Table S2 Diagnostic performance of AI , individual radiologists, and AI-assisted radiologists taking PPE as the evaluation standard.

| **Method** | **PPE** | | **Sensitivity**  **95% CI** | **p value** | **Specificity**  **95% CI** | **p value** | **Accuracy**  **95% CI** | **p value** | **AUC**  **95% CI** | **p value** |
| --- | --- | --- | --- | --- | --- | --- | --- | --- | --- | --- |
|  | **Malignancy** | **Benign** |  |  |  |  |  |  |  |  |
| **AI** |  | | | | | | | | | |
| Malignancy | 308 | 56 | 0.824 (0.785, 0.862) |  | 0.835 (0.796, 0.875) |  | 0.829 (0.802, 0.857) |  | 0.829 (0.802, 0.857) |  |
| Benign | 66 | 284 |  |  |  |  |  |  |  |  |
| **Junior radiologist** |  | | | | | | | | | |
| Malignancy | 295 | 95 | 0.789 (0.747, 0.830) | 0.096* | 0.721 (0.673, 0.768) | **<0.001*** | 0.756 (0.725, 0.788) | **<0.001*** | 0.755 (0.723, 0.786) | **<0.001*** |
| Benign | 79 | 245 |  |  |  |  |  |  |  |  |
| **Senior radiologist** |  | | | | | | | | | |
| Malignancy | 299 | 60 | 0.799 (0.759, 0.840) | 0.245* | 0.824 (0.783, 0.864) | 0.555* | 0.811 (0.782, 0.840) | 0.370* | 0.811 (0.783, 0.840) | 0.378* |
| Benign | 75 | 280 |  |  |  |  |  |  |  |  |
| **Junior radiologist+ AI** |  | | | | | | | | | |
| Malignancy | 298 | 63 | 0.797 (0.756, 0.838) | 0.708^ | 0.815 (0.773, 0.857) | **<0.001^** | 0.805 (0.776, 0.834) | **0.025^** | 0.806 (0.777, 0.835) | **0.020^** |
| Benign | 76 | 277 |  | 0.900^#^ |  | 0.665^#^ |  | 0.788^#^ |  | 0.782^#^ |
| **Senior radiologist+AI** |  | | | | | | | | | |
| Malignancy | 297 | 53 | 0.794 (0.753, 0.835) | 0.802^ | 0.844 (0.806, 0.882) | 0.296^ | 0.818 (0.790, 0.846) | 0.743^ | 0.819 (0.791, 0.847) | 0.711^ |
| Benign | 77 | 287 |  |  |  |  |  |  |  |  |
| **Arbitration Committee** |  | | | | | | | | | |
| Malignancy | 295 | 43 | 0.789 (0.747, 0.830) |  | 0.865 (0.829, 0.900) |  | 0.825 (0.797, 0.853) |  | 0.827 (0.799, 0.854) |  |
| Benign | 78 | 294 |  |  |  |  |  |  |  |  |

PPE postoperative Pathological Examinations; CI confidence interval; AUC area under the receiver operating characteristic curve; AI artificial intelligence; *p values to compare radiologists with the AI system; ^p values to compare radiologists with and without assistance of AI;  ^#^p values to compare junior radiologists with assistance of AI to senior radiologists alone.

Table S3 Medical Center 1: diagnostic performance of AI , individual radiologists, and AI-assisted radiologists, evaluated against 2e diagnostic criteria.

| **Method** | **2e diagnostic criteria** | | **Sensitivity**  **95% CI** | **p value** | **Specificity**  **95% CI** | **p value** | **Accuracy**  **95% CI** | **p value** | **AUC**  **95% CI** | **p value** |
| --- | --- | --- | --- | --- | --- | --- | --- | --- | --- | --- |
|  | **Malignancy** | **Benign** |  |  |  |  |  |  |  |  |
| **AI** |  | | | | | | | | | |
| Malignancy | 255 | 47 | 0.889 (0.852, 0.925) |  | 0.938 (0.920, 0.955) |  | 0.924 (0.908, 0.940) |  | 0.913 (0.896, 0.930) |  |
| Benign | 31 | 706 |  |  |  |  |  |  |  |  |
| **Junior radiologist** |  | | | | | | | | | |
| Malignancy | 246 | 88 | 0.861 (0.821, 0.901) | 0.055* | 0.883  (0.860, 0.906) | **<0.001*** | 0.877  (0.857, 0.897) | **<0.001*** | 0.872  (0.851, 0.892) | **0.002*** |
| Benign | 40 | 665 |  |  |  |  |  |  |  |  |
| **Senior radiologist** |  | | | | | | | | | |
| Malignancy | 248 | 41 | 0.861 (0.821, 0.901) | 0.055* | 0.944 (0.928, 0.961) | 0.521* | 0.921 (0.905, 0.937) | 0.806* | 0.902 (0.884, 0.920) | 0.403* |
| Benign | 38 | 712 |  |  |  |  |  |  |  |  |
| **Junior radiologist+AI** |  | | | | | | | | | |
| Malignancy | 241 | 45 | 0.836 (0.793, 0.879) | 0.121^ | 0.939 (0.922, 0.956) | **<0.001^** | 0.910 (0.893, 0.928) | **0.013^** | 0.888 (0.868, 0.907) | 0.270^ |
| Benign | 45 | 708 |  | 0.121^#^ |  | 0.605^#^ |  | 0.385^#^ |  | 0.269^#^ |
| **Senior radiologist+AI** |  | | | | | | | | | |
| Malignancy | 248 | 33 | 0.861 (0.821, 0.901) | 1.000^ | 0.955 (0.940, 0.970) | 0.268^ | 0.929 (0.913, 0.944) | 0.505^ | 0.908 (0.890, 0.925) | 0.679^ |
| Benign | 38 | 720 |  |  |  |  |  |  |  |  |

CI confidence interval; AUC area under the receiver operating characteristic curve; AI artificial intelligence; *p values to compare radiologists with the AI system; ^p values to compare radiologists with and without assistance of AI;  ^#^p values to compare junior radiologists with assistance of AI to senior radiologists alone.

Table S4 Medical Center 2: diagnostic performance of AI , individual radiologists, and AI-assisted radiologists, evaluated against 2e diagnostic criteria.

| **Method** | **2e diagnostic criteria** | | **Sensitivity**  **95% CI** | **p value** | **Specificity**  **95% CI** | **p value** | **Accuracy**  **95% CI** | **p value** | **AUC**  **95% CI** | **p value** |
| --- | --- | --- | --- | --- | --- | --- | --- | --- | --- | --- |
|  | **Malignancy** | **Benign** |  |  |  |  |  |  |  |  |
| **AI** |  | | | | | | | | | |
| Malignancy | 72 | 24 | 0.873 (0.800, 0.947) |  | 0.922 (0.894, 0.950) |  | 0.913 (0.886, 0.940) |  | 0.898 (0.869, 0.927) |  |
| Benign | 8 | 322 |  |  |  |  |  |  |  |  |
| **Junior radiologists** |  | | | | | | | | | |
| Malignancy | 76 | 121 | 0.924 (0.866, 0.982) | **0.014*** | 0.651 (0.601, 0.701) | **<0.001*** | 0.702 (0.658, 0.745) | **<0.001*** | 0.788 (0.749, 0.827) | **<0.001*** |
| Benign | 4 | 225 |  |  |  |  |  |  |  |  |
| **Senior radiologists** |  | | | | | | | | | |
| Malignancy | 75 | 32 | 0.911 (0.849, 0.974) | 0.073* | 0.899 (0.867, 0.931) | 0.238* | 0.901 (0.873, 0.930) | 0.555* | 0.905 (0.877, 0.933) | 0.715* |
| Benign | 5 | 314 |  |  |  |  |  |  |  |  |
| **Junior radiologists+AI** |  | | | | | | | | | |
| Malignancy | 74 | 31 | 0.899 (0.832, 0.965) | 0.193^ | 0.902 (0.870, 0.934) | **<0.001^** | 0.901 (0.873, 0.930) | **<0.001^** | 0.900 (0.872, 0.929) | **<0.001^** |
| Benign | 6 | 315 |  | 0.528^#^ |  | 0.888^#^ |  | 1.000^#^ |  | 0.810^#^ |
| **Senior radiologists+AI** |  | | | | | | | | | |
| Malignancy | 74 | 24 | 0.899 (0.832, 0.965) | 0.528^ | 0.922 (0.894, 0.950) | 0.238^ | 0.918 (0.892, 0.944) | 0.403^ | 0.910 (0.883, 0.938) | 0.793^ |
| Benign | 6 | 322 |  |  |  |  |  |  |  |  |

CI confidence interval; AUC area under the receiver operating characteristic curve; AI artificial intelligence; *p values to compare radiologists with the AI system; ^p values to compare radiologists with and without assistance of AI;  ^#^p values to compare junior radiologists with assistance of AI to senior radiologists alone.

Table S5 Medical Center 3: diagnostic performance of AI , individual radiologists, and AI-assisted radiologists, evaluated against 2e diagnostic criteria.

| **Method** | **2e diagnostic criteria** | | **Sensitivity**  **95% CI** | **p value** | **Specificity**  **95% CI** | **p value** | **Accuracy**  **95% CI** | **p value** | **AUC**  **95% CI** | **p value** |
| --- | --- | --- | --- | --- | --- | --- | --- | --- | --- | --- |
|  | **Malignancy** | **Benign** |  |  |  |  |  |  |  |  |
| **AI** |  | | | | | | | | | |
| Malignancy | 184 | 42 | 0.839 (0.790, 0.887) |  | 0.936 (0.916, 0.955) |  | 0.910 (0.890, 0.929) |  | 0.887 (0.866, 0.909) |  |
| Benign | 35 | 570 |  |  |  |  |  |  |  |  |
| **Junior radiologists** |  | | | | | | | | | |
| Malignancy | 162 | 70 | 0.731 (0.673, 0.789) | **<0.001*** | 0.887 (0.861, 0.912) | **0.001*** | 0.845 (0.820, 0.869) | **<0.001*** | 0.809 (0.782, 0.835) | **<0.001*** |
| Benign | 57 | 542 |  |  |  |  |  |  |  |  |
| **Senior radiologists** |  | | | | | | | | | |
| Malignancy | 181 | 35 | 0.812 (0.760, 0.863) | 0.149* | 0.942 (0.924, 0.961) | 0.575* | 0.907 (0.888, 0.927) | 0.865* | 0.877 (0.855, 0.899) | 0.521* |
| Benign | 38 | 577 |  |  |  |  |  |  |  |  |
| **Junior radiologists+AI** |  | | | | | | | | | |
| Malignancy | 171 | 55 | 0.771 (0.716, 0.826) | 0.057^ | 0.911 (0.888, 0.934) | 0.095^ | 0.874 (0.851, 0.896) | 0.090^ | 0.841 (0.816, 0.866) | **0.030**^ |
| Benign | 48 | 557 |  | **0.043^#^** |  | **0.014^#^** |  | **0.028^#^** |  | **0.036^#^** |
| **Senior radiologists+AI** |  | | | | | | | | | |
| Malignancy | 181 | 30 | 0.812 (0.760, 0.863) | 1.000^ | 0.951 (0.934, 0.968) | 0.456^ | 0.913 (0.894, 0.932) | 0.668^ | 0.881 (0.859, 0.903) | 0.797^ |
| Benign | 38 | 582 |  |  |  |  |  |  |  |  |

CI confidence interval; AUC area under the receiver operating characteristic curve; AI artificial intelligence; *p values to compare radiologists with the AI system; ^p values to compare radiologists with and without assistance of AI;  ^#^p values to compare junior radiologists with assistance of AI to senior radiologists alone.

Table S6 Six representative situations of the AI system and the radiologists in the diagnosis results.

| **Representative situation** | **Number of thyroid nodules** |
| --- | --- |
| Both the AI system and senior radiologists misdiagnosed "benign" as "malignant", and the final result was "benign". | 81 |
| Both the AI system and senior radiologists misdiagnosed "malignant" as "benign", and the final result was "malignant". | 59 |
| The AI system diagnosed as "malignant", which was changed from "benign" to "malignant" by junior radiologists after referring to AI, and the final result was "malignant". | 32 |
| The AI system diagnosed as "malignant", which was changed from "benign" to "malignant" by junior radiologists after referring to AI, and the final result was "benign". | 17 |
| The AI system diagnosed as "benign", which was changed from "malignant" to "benign" by junior radiologists after referring to AI, and the final result was "malignant". | 14 |
| The AI system was diagnosed as "benign", which was changed from "malignant" to "benign" by junior radiologists after referring to AI, and the final result was "benign". | 161 |

Table S7 Nodules in the thyroid isthmus: diagnostic performance of AI , individual radiologists, and AI-assisted radiologists, evaluated against 2e diagnostic criteria.

| **Method** | **2e diagnostic criteria** | | **Sensitivity**  **95% CI** | **p value** | **Specificity**  **95% CI** | **p value** | **Accuracy**  **95% CI** | **p value** | **AUC**  **95% CI** | **p value** |
| --- | --- | --- | --- | --- | --- | --- | --- | --- | --- | --- |
|  | **Malignancy** | **Benign** |  |  |  |  |  |  |  |  |
| **AI** |  | | | | | | | | | |
| Malignancy | 47 | 7 | 0.810 (0.709, 0.911) |  | 0.904 (0.837, 0.972) |  | 0.863 (0.804, 0.922) |  | 0.857 (0.797, 0.917) |  |
| Benign | 11 | 66 |  |  |  |  |  |  |  |  |
| **Junior radiologist** |  | | | | | | | | | |
| Malignancy | 44 | 15 | 0.759 (0.648, 0.869) | 0.308* | 0.795 (0.702, 0.887) | **0.012*** | 0.779 (0.708, 0.850) | 0.075* | 0.777 (0.705, 0.848) | **0.031*** |
| Benign | 14 | 58 |  |  |  |  |  |  |  |  |
| **Senior radiologist** |  | | | | | | | | | |
| Malignancy | 46 | 7 | 0.793 (0.689, 0.897) | 0.726* | 0.904 (0.837, 0.972) | 1.000* | 0.855 (0.795, 0.915) | 0.859* | 0.849 (0.787, 0.910) | 0.777* |
| Benign | 12 | 66 |  |  |  |  |  |  |  |  |
| **Junior radiologist+AI** |  | | | | | | | | | |
| Malignancy | 46 | 6 | 0.793 (0.689, 0.897) | 0.503^ | 0.918 (0.855, 0.980) | **0.004^** | 0.863 (0.804, 0.922) | 0.075**^** | 0.855 (0.795, 0.916) | **0.016**^ |
| Benign | 12 | 67 |  | 1.000^#^ |  | 0.697^#^ |  | 0.859^#^ |  | 0.813^#^ |
| **Senior radiologist+AI** |  | | | | | | | | | |
| Malignancy | 45 | 6 | 0.776 (0.669, 0.883) | 0.734^ | 0.918 (0.855, 0.980) | 0.697^ | 0.855 (0.795, 0.915) | 1.000^ | 0.847 (0.785, 0.909) | 0.915^ |
| Benign | 13 | 67 |  |  |  |  |  |  |  |  |

CI confidence interval; AUC area under the receiver operating characteristic curve; AI artificial intelligence; *p values to compare radiologists with the AI system; ^p values to compare radiologists with and without assistance of AI;  ^#^p values to compare junior radiologists with assistance of AI to senior radiologists alone.

.
